# Preferential Lentiviral Targeting of Astrocytes in the Central Nervous System

**DOI:** 10.1371/journal.pone.0076092

**Published:** 2013-10-02

**Authors:** Michael Fassler, Itai Weissberg, Nitzan Levy, Felipe Diaz-Griffero, Alon Monsonego, Alon Friedman, Ran Taube

**Affiliations:** 1 The Shraga Segal Department of Microbiology, Immunology and Genetics, Faculty of Health Sciences, Ben-Gurion University of the Negev, Be’er Sheva, Israel; 2 Departments of Physiology and Cell Biology, Cognitive and Brain Sciences, Zlotowski Centre for Neuroscience, Ben-Gurion University of the Negev, Be’er Sheva, Israel; 3 Department of Microbiology and Immunology, Albert Einstein College of Medicine, Bronx, New York, United States of America; Emory University, United States of America

## Abstract

The ability to visualize and genetically manipulate specific cell populations of the central nervous system (CNS) is fundamental to a better understanding of brain functions at the cellular and molecular levels. Tools to selectively target cells of the CNS include molecular genetics, imaging, and use of transgenic animals. However, these approaches are technically challenging, time consuming, and difficult to control. Viral-mediated targeting of cells in the CNS can be highly beneficial for studying and treating neurodegenerative diseases. Yet, despite specific marking of numerous cell types in the CNS, *in vivo* selective targeting of astrocytes has not been optimized. In this study, preferential targeting of astrocytes in the CNS was demonstrated using engineered lentiviruses that were pseudotyped with a modified Sindbis envelope and displayed anti-GLAST IgG on their surfaces as an attachment moiety. Viral tropism for astrocytes was initially verified *in vitro* in primary mixed glia cultures. When injected into the brains of mice, lentiviruses that displayed GLAST IgG on their surface, exhibited preferential astrocyte targeting, compared to pseudotyped lentiviruses that did not incorporate any IgG or that expressed a control isotype IgG. Overall, this approach is highly flexible and can be exploited to selectively target astrocytes or other cell types of the CNS. As such, it can open a window to visualize and genetically manipulate astrocytes or other cells of the CNS as means of research and treatment.

## Introduction

The importance of the *neurovascular unit* in the preservation of the normal functions of the central nervous system (CNS) is well documented. Cross talk between different cell types within this unit is critical, and its dysfunction has been linked to several human pathologies of the brain [1–3]. Specifically, interactions between neurons and glia cells are important in modulating brain functions under normal and disease conditions. Astrocytes are also key regulators in the brain, playing significant roles in physiological processes, such as energy metabolism, homeostasis of ions, and synaptic cross talk. As such, astrocyte dysfunctions may promote neurodegenerative pathologies [4–9]. However, our understanding of the role of astrocytes in establishing neurological disorders is not clear, since current knowledge derives mainly from *in vitro* analysis and is severely hampered by the lack of *in vivo* models.

To better elucidate the role of astrocytes in promoting both normal and pathological processes, efficient gene transfer and gene manipulation of these cells is highly beneficial. However, gene delivery into astrocytes (and other cells of the CNS) remains challenging, due to the complexity of the tissue. The presence of the blood-brain barrier [10] and the lack of tools to manipulate gene expression *in vivo* in specific cells, also contribute to the poor progress in understanding the roles of astrocytes in the CNS [11,12]. Several approaches have attempted to specifically mark and manipulate genes in cells of the CNS. The expression of inert reporter proteins or indicators in well-defined sub-populations of cells of the CNS has made an important contribution to these attempts [13–15]. In addition, Cre-loxP mice have also been used to facilitate genetic manipulation in specific cells [16]. Finally, cell-specific promoters have also been used for controlling gene expression in specific cells in the CNS [17]. For example, the GFAP promoter has been well characterized and has been extensively and successfully utilized to efficiently and selectively drive long-lasting transgene expression both *in vitro* and *in vivo* [18]. However, the use of other cell-specific promoters may be limited, as not many have been characterized, and in some cases, tissue-specific expression is difficult to maintain [19–23].

Viral vectors that carry a transgene of interest and that can be delivered into defined areas and cells in the CNS is also a well-established practice [24,25]. Among those vectors that are frequently exploited, lentiviral vectors are highly attractive. They are easy to manipulate, transduce both dividing and non-dividing cells, support sustained expression of transgenes, and have relatively large packaging capacity and low immune toxicity [25–28]. Initial *in vitro* studies of the feasibility of lentiviral vectors to transduce cells of the CNS were performed by Naldini et al., who demonstrated efficient transduction of neurons with prolonged transgene expression [29,30]. However, those studies exploited lentivectors that had been pseudotyped with glycoproteins from the vesicular stomatitis virus (VSV-G), thus displayed non-selective and broad tropism towards a wide variety of cells. VSV-G pseudotyped lentiviruses have been commonly used for *in-vivo* gene transfer applications, but they only facilitate non-specific marking of cells [31]. To overcome this problem and to achieve specific targeting towards target cells, other viral glycoproteins have been used instead of the VSV-G glycoprotein. Lentiviral pseudotyping with rabies G glycoprotein, paramyxovirus, or measles have all been utilized and demonstrated a shift in the particle’s ability to change its cell specificity [32–34]. Other glycoproteins from Ebola virus, *arenavirus*, lymphocytic choriomeningitis virus (LCMV), Mokola virus (MV), Moloney murine leukemia virus (MuLV), and Ross River virus (RRV) have also been evaluated for their ability to transduce different cells of the brain, among them astrocytes [35,36]. A different approach that may further improve viral selective cell tropism, utilizes particles that have been pseudotyped with modified viral glycoproteins, which retain fusogenic activity but lose their ability to bind to target cells. In these cases, a binding moiety has to be supplemented. Such tools include the modified VSV-G (VSV-GS), which mediates only fusion to cell specific ligands [37]. Attachment of the viral particle to its target cells may be also acquired via soluble ASLV receptor, which is conjugated to viral particles that display the ASLV envelope [37]. Binding moieties in the form of IgG, single-chain fragments (scFv), specific ligands, or peptides that are incorporated on the surface of the particles are also commonly used [38–40]. In these approaches, lentiviral targeting has been applied successfully *in-vivo* on primary endothelial cells [41,42], dendritic cells [43], macrophages [44], hematopoietic progenitor cells [45,46] and lymphocytes [47,48]. Cell targeting has also been achieved by incorporating scFv against the AMPA glutamate receptor subunits GluA2 and GluA4. Expression of these binding moieties on surfaces of viral particles have been utilized for specific viral attachment to neurons, endothelial cells and hematopoietic progenitors [49]. Finally, cell-specific gene delivery by lentiviral vectors has been providing new options for efficient therapy by modulating immune cells [50]. Nevertheless, *in-vivo* targeting of astrocytes in the CNS has not yet been successfully demonstrated.

In this study, we tested the ability of engineered lentiviruses that display a specific GLAST IgG on their surface to preferentially target astrocytes both *in vitro* and *in vivo*. Reporter viral particles that expressed the Zoanthus Green Fluorescent (ZsGreen) gene were pseudotyped with a sindbis modified envelope that could not mediate viral attachment due to mutations in the E2 envelope binding region but did preserve the E1 fusogenic moiety (sindMu-ZsGreen) [51]. Attachment of viral particles to astrocytes was complemented by a soluble IgG that bound the glutamate transporter GLAST (also known as EAAT1 and ASCA-1; astrocyte cell surface antigen-1), a unique surface astrocyte marker. IgG targeting moieties were incorporated onto lentiviral sindMu-ZsGreen particles via a ZZ motif that bound the human Fc region and inserted in-frame into the envelope gene. This delivery application was successfully exploited *in vitro* by transducing primary astrocytes isolated from mixed glia cultures. Significantly, it was also validated *in vivo* following injection of recombinant viruses into the brain and mark astrocytes. We concluded that recombinant lentiviruses that were pseudotyped with a modified sindbis envelope and displayed GLAST IgG as a binding moiety could potentially mark astrocytes and modify viral tropism for these cells. Such an approach could be used for analyzing gene function in astrocytes and for tracing or imaging studies in the CNS during brain development under normal or pathological conditions.

## Materials and Methods

### Cells

Human embryonic kidney 293T (HEK 293T) cells were obtained from the American Type Culture Collection. Cells were maintained in complete DMEM. Primary mixed glial cell cultures were prepared from the cerebral cortices of one-day-old C57BL/6 mice (Harlan, Israel). Brain tissue was digested by incubation with trypsin 2.5% supplemented with 0.5 mg/mL DNase I (Sigma), followed by treatment with 5 mg/mL DNase I. Digested tissue was then passed through a thin pipette several times to ensure homogenesis. The cells were then re-suspended in DMEM supplemented with 10% FBS, 4 mM L-glutamine, penicillin (100 U/mL), streptomycin (1 µg/ml), nystatin (2.5 U/mL), 10 mM HEPES, 1 mM sodium pyruvate, 10 mM nonessential amino acids and 50 µM β-mercapthoethanol; seeded into poly-D-lysine-coated flasks; and kept at 37°C in a humidified atmosphere of 5% CO_2_ and 95% air. Growth medium was replenished after 24 h and every 2-3 days.

### Lentivirus Production

Self-inactivated lentiviruses were generated in 293T by the calcium phosphate (CaPO_4_) transfection method. The DNA transfection mixture included HIV packaging expression plasmids (Gag, Tat, Rev), the ZsGreen lenti-transvector under the control of the CMV promoter (provided by the Mulligan lab, Harvard Medical School), and the indicated envelope expression plasmid. Viruses were pseudotyped with either the VSV-G glycoprotein for nonspecific transduction or the modified sindbis envelope (sindMu), which only mediated specific viral targeting (kindly provided by the Chen lab, UCLA). 48h post transfection, the supernatant containing the viral particles was collected, filtered, and concentrated by ultracentrifugation on a 20% sucrose cushion at 25,000 rpm (112,700 rcf) for 2 h at 4°C. High-tittered viral stocks were stored at -80°C until transduction experiments were performed. Viral titers for VSV-G pseudotyped reporter viruses were calculated by transducing 293T cells with increasing amounts of the virus and monitoring viral ZsGreen expression 48h later by FACS (FACS Calibur, BD Biosciences). VSV-G pseudotyped lentiviral titers were calculated as 5×10^8^/ml. Titers for the sindMu pseudotype lentiviruses were quantified by a p24 ELISA and set to 500 ng p24/µl (Abbot).

### Incorporation of IgG on lentiviruses

Incorporation of the monoclonal mouse anti-GLAST IgG or other IgG (Miltenyi Biotec, Germany) onto the sindMu pseudotyped lentivirus was performed by incubating sindMu-ZsGreen viral particles with the soluble IgG on ice for 1 h before transduction. Antibody concentrations used for *in-vitro* and *in vivo* experiments were 1.5µg/ml and 2 µg/ml, respectively. Optimal IgG incorporation was analyzed *in vitro* on primary glia cultures. Serial dilutions (0.5, 1, 1.5, 2 and 3 µg/ml antibody) were tested for optimal viral attachment to target cells. Transduction experiments were performed by incubating the viruses for 4.5h on cells, followed by washing with PBS, and adding new cell medium. Cells were then incubated for 48h and analyzed by FACS for ZsGreen expression.

### *In vitro* targeting of astrocytes from mixed primary glia cultures by sindMu-ZsGreen/GLAST IgG

Primary glia cells were transduced with engineered sindMu-ZsGreen/GLAST IgG lentivirus. Lentiviruses were diluted to 100 ng p24/µl stock, and 5 µl were used for transduction. As control, cells were also transduced with sindMu-ZsGreen lentiviruses that did not incorporate an IgG targeting moiety or particles that incorporated isotype IgG (Miltenyl Biotech). 48h post transduction, cells were fixed with 4% paraformaldehyde for 1h, and washed twice with PBS-Tween-0.05%. Cells were then permeabilized and blocked for 30 minutes with a blocking solution containing 0.05% Triton X-100 (Sigma). To detect primary astrocytes, cells were incubated with a monoclonal anti-GFAP conjugated to APC (Bio-legend) at 4°C for 1 h. Cells were then washed and subjected to FACS analysis for monitoring ZsGreen and GFAP-APC expression. For control of non-specific transduction, sindMu-ZsGreen lentivirus were pseudotyped with VSV-G envelope. Cultures grown *in vitro* were also stained for microglia marker using primary CD11b antibody (Bio-legend) and analyzed by FACS.

### *In vivo* injections

FVB-N and ICR mice were purchased from Harlan (Israel). All mice used in this study were bred and maintained in the local animal care facility, approved by the Institutional Animal Care and Use Committee (IACUC) of Ben-Gurion University of the Negev (BGU). All surgical and experimental procedures were reviewed and approved by the Institutional Animal Care and Use Committee (IACUC) of Ben-Gurion University of the Negev (BGU’s IACUC) according to specified protocols that aim to ensure animal welfare and reduce suffering. All mice were housed under a 12h light:dark cycle with food and water ad libitum. 2-3 month-old mice were deeply anesthetized with an i.m. injection of 90-120 mg/kg ketamine (Ketaset, Fort Dodge, Iowa, USA) and 6 mg/kg xylazine (XYL-M2, VMD, Belgium). sindMu-ZsGreen/IgG lentiviruses (diluted to 200 ng p24/µl) were injected stereotacticly with a 10µl syringe (1701RN, Hamilton, Switzerland) held in a stereotaxic syringe pump (Chemyx Nanojet syringe pump, Texas, USA) into the dorsal hippocampus, CA1 (2.1 mm caudal, 2 mm lateral and 1.5–2.5 mm ventral to bregma) or to the thalamus/ventricle of the mouse brain (+0.5 mm caudal, 0.75 mm lateral and 2.5-2.5 mm ventral to bregma). Typical viral injection volumes were 4 µl at a rate of 0.5 µl/min [52,53].

### Immunofluorescence of cells following *in vivo* lentiviral injection

14 days post lentiviral injection, mice were anesthetized and perfused with 100 ml/kg body weight of 4% paraformaldehyde in 0.1 M PBS. Mice were decapitated, and the brains were carefully removed from the skull and kept in a fixative solution containing 4% paraformaldehyde at 4°C overnight. Samples were further incubated with 10% sucrose in PBS for 24h, followed by an additional 24h of incubation in 30% sucrose, and then frozen in O.C.T compound (Tissue-Tek O.C.T, Sakura, USA) by liquid nitrogen and stored at -80°C. Thin (40 µM) coronal sections were sliced with a cryostat (CM1850 LEICA Microsystems, Germany). For staining of astrocytes *in vivo*, sections were blocked with 10% BSA and stained for anti-glial fibrillary acidic protein (GFAP) expression with a primary anti-GFAP rabbit polyclonal antibody at 4°C overnight. Brain sections were washed with PBS-Tween 0.05% and stained with anti-rabbit Alexa-fluor 546 secondary antibody for an additional 1h at room temperature. For staining neurons, the same stain protocol was performed, using mouse anti-NeuN primary antibody and anti-mouse Alexa flour 546 secondary antibody. To verify lentiviral transduction, brain sections were also analyzed by confocal microscopy for ZsGreen expression (FlowView FV1000, Olympus, Japan) [54]. For each set of conditions, the numbers of ZsGreen-NeuN or ZsGreen-GFAP positive cells were determined on the images (six images per section, three sections, three animals) using a Flow View FV1000, Olympus imaging microscope equipped with automated motorized stage and an image acquisition and analysis system (ImageJ). Co-localization immuno-staining percentage was then expressed in arbitrary index units.

## Results

### Generation of recombinant lentiviruses for targeting astrocytes

Our aim was to establish a tool for specific targeting of astrocytes, using lentiviral vectors. To this end, recombinant reporter lentiviruses that expressed ZsGreen under the control of the CMV promoter were produced in 293T cells by expressing the required HIV expression plasmids: Gag-Pol, Rev, Tat, the ZsGreen reporter transgene, as well as the indicated envelope. ZsGreen reporter lentiviruses were pseudotyped with two types of envelope glycoprotein; (i) a sindbis modified glycoprotein (SindMu), which preserves the E1 fusogenic region of the sindbis viral envelope but does not mediate viral attachment due to mutations in the E2 glycoprotein-binding motif; (ii) a VSV-G glycoprotein, which exhibits non-specific and broad transduction efficiencies. 48 hours post transfection, the supernatant containing the lentivirus was collected, cleared and concentrated by ultracentrifugation. sindMu-ZsGreen lentiviral stocks were stored at -80°C until used for transduction of target cells. Soluble IgGs were used as attachment moieties to target cells. IgGs were incorporated on the surface of sindMu-ZsGreen lentivirus by incubating viral particles on ice for 1h with optimal IgG concentrations (2 µg/ml). Soluble IgG incorporation on viral particles was obtained via a ZZ sequence (a tandem repeated, mutated domain B derived from the antibody-binding protein-A of *Staphylococcus aureus*), which was inserted in-frame into the modified sindbis viral glycoprotein [38]. ZZ motif served as a docking motif for binding IgG to the surfaces of the viral particles [38,55,56]. For astrocyte specific targeting, anti-GLAST-1 IgG was used (sindMu-ZsGreen/GLAST IgG). GLAST is a glutamate transporter that is predominantly expressed on astrocytes and can thus be used as a binding moiety for recombinant lentiviruses [57,58]. As control for specificity of viral particles, isotype IgG was also incorporated on the surface of sindMu-ZsGreen lentiviruses (SindMu-ZsGreen/IgG isotype). Titers of lentiviruses were determined by p24 ELISA. VSV-G-ZsGreen titers were also calculated by FACS, following transduction of 293T cells and analysis of ZsGreen expression 48h post transduction. In that assay, viral titers of VSV-G-ZsGreen lentivirus were high, reaching up to 5×10^8^ particles/ml. Overall, equivalent amounts (5 µl of 100 ng p24/µl) of the VSV-G ZsGreen or sindMu-ZsGreen/ IgG lentiviral stocks were used to transduce target cells *in vitro*.

### *In vitro* targeting of primary astrocytes in a mixed glia culture

**Figure 1:**
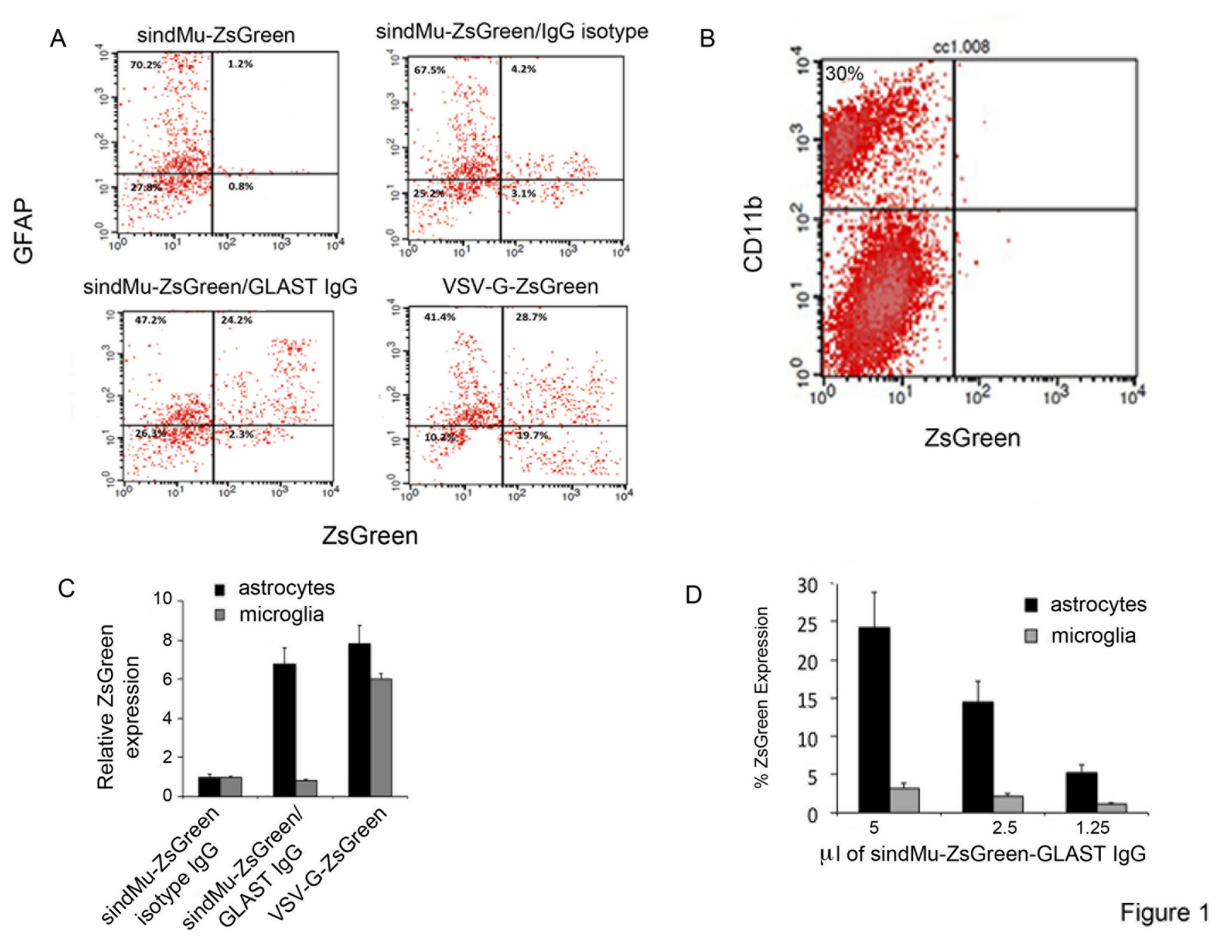
Preferential *in vitro* targeting of primary astrocytes by engineered lentiviruses sindMu-ZsGreen/GLAST IgG. **A**. *In* *vitro* targeting of astrocytes by lentiviruses – mixed primary glia cells isolated from the brains of one day old mice and were cultured *in* *vitro*. Cultures were transduced with the indicated engineered lentiviruses, and 48h post transduction cells were harvested and stained for GFAP to specifically detect astrocytes. Cells were then analyzed by FACS for ZsGreen expression and for specific GFAP staining. **Panel (a)** - transduction of cells with lentiviruses that did not incorporate IgG (sindMu-ZsGreen). **Panel (b)** - transduction of cells with lentiviruses that incorporated an IgG isotype (sindMu-ZsGreen/IgG isotype). **Panel (c)** - transduction of cells with lentiviruses that incorporated soluble GLAST IgG (sindMu-ZsGreen/GLAST IgG) and demonstrated preferential targeting for astrocytes. **Panel (d)** - transduction of cells with lentiviruses that were pseudotyped with VSV-G and exhibited broad non-specific transduction to cells in the culture (VSV-G-ZsGreen). **B**. FACS analysis demonstrating the composition of the mixed microglia culture. Cells of mixed microglia cultures were transduced with sindMu-ZsGreen/IgG isotype and stained for CD11b antigen as specific marker of microglia. Cells were then washed and analyzed for ZsGreen expression and CD11b staining by FACS. **C**. A summary of quantitation of the *in* *vitro* targeting of mixed microglia cell cultures. Cells were transduced with the indicated lentiviruses. 48h post transduction, cells were harvested and stained for astrocyte staining (GFAP) or microglia (CD11b). Cells were then analyzed by FACS for ZsGreen expression and for cell specific antigen. Values are presented relatively to the transduction efficiencies by the sindMu-ZsGreen/isotype IgG lentivirus. Results are representative of the means of triplicate wells; error bars show the standard deviation of the means. **D**. Increasing amounts of the sindMu-ZsGreen/GLAST IgG lentivirus were used to transduce 3x10^5^ cells/well primary glia cultures. 48h post transduction, cells were harvested and stained for astrocyte staining (GFAP) or microglia (CD11b). Cells were then analyzed by FACS for ZsGreen expression and for cell specific antigen. Results are representative of the means of triplicate wells; error bars show the standard deviation of the means.

Initially, the leniviral targeting approach was validated *in vitro*. A mixed glia cell culture, which consisted mainly of astrocytes and microglia, was isolated from the brains of young mice. The composition of the cell populations in the culture was determined by staining cells for the expression of glial fibrillary acidic protein (GFAP) astrocyte marker, and analysis by FACS. Cultures were also stained for microglia marker with CD11b antibodies. Close to 70% of the cells were marked as astrocytes, as measured by GFAP specific staining (Figure 1A). In contrast, only 30% were stained positively for CD11b (Figure 1B).

To examine the tropism of sindMu-ZsGreen/GLAST IgG lentiviruses for astrocytes *in vitro*, cells were transduced with this recombinant reporter lentiviruses (estimated MOI of 5). sindMu-ZsGreen/IgG isotype lentiviruses, or sindMu-ZsGreen that did not incorporate IgG were used for control for transductions specificity (Figure 1A). To demonstrate non-specific and broad transduction of cells, mixed cultures were also transduced with lentiviruses that were pseudotyped with the VSV-G glycoprotein (VSV-G-ZsGreen; Figure 1A). 48h post transduction, cells were harvested and stained for GFAP (astrocytes) or CD11b (microglia) cell markers. The efficiency of selective marking of astrocytes *versus* microglia was monitored by FACS by analyzing ZsGreen expression and GFAP or CD11b staining (Figure 1A and 1B). Importantly, lentiviruses had no effect on cell viability throughout all the experiments.

Preferential tropism of the sindMu-ZsGreen/GLAST IgG lentivirus for astrocytes was indeed detected, as approximately 33.9% of total astrocytes (i.e., 24.2/71.4% of total astrocyte cells; Figure 1A) were positively marked with ZsGreen and GFAP. In contrast, only 8% of the microglia cells (i.e., 2.3/28.6% of total GFAP negatively stained microglia cells; Figure 1A) were marked with the sindMu-ZsGreen/GLAST IgG lentivirus. Furthermore, 10% of microglia (i.e., 3.1/28.3% of total microglia cells; close to background levels) and 5.8% of astrocytes (i.e., 4.2/71.7% of total cells) were targeted by the sindMu-ZsGreen/IgG isotype lentivirus that incorporated non-specific IgG (Figure 1A). Similarly, low transduction efficiencies were detected when sindMu-ZsGreen lentiviruses that did not display IgG on their surfaces were used (Figure 1A). When cells in the mixed culture were transduced with lentiviral reporter viruses that were pseudotyped with VSV-G (VSV-G-ZsGreen), high transduction efficiencies of both microglia (65.8%; 19.7/29.9% of total microglia cells in the culture) and astrocytes (40%; 28.7/70.2% of total astrocyte cells in the culture) were observed (Figure 1A). These results confirmed the relatively broad tropism of VSV-G pseudotyped lentivirus for various cells in the culture and high transduction rates. A summary of the FACS analysis transduction experiments, relative to the control isotype IgG sindMu-ZsGreen lentivirus, clearly demonstrated that the sindMu-ZsGreen/GLAST IgG lentiviral-targeting approach exhibited higher tropism for astrocytes *in vitro* (Figure 1C).

To further confirm the specificity of the sindMu-ZsGreen/GLAST IgG for astrocytes, increasing MOIs of this specific lentivirus were used to transduce primary microglia mixed cultures (Figure 1D). MOI were calculated as the ratio between virus amounts and cell numbers, and was estimated as MOI of 5. As shown, as MOI values of sindMu-ZsGreen/GLAST IgG lentivirus decreased, (5; 2.5 1.25) the ability of sindMu-ZsGreen/GLAST IgG lentivirus to transduce astrocytes was reduced. Importantly, transduction efficiencies of the sindMu-ZsGreen/GLAST IgG to transduce microglia did not change at different MOI values and remained at background levels (Figure 1D). These results confirm the specificity of the sindMu-ZsGreen/GLAST IgG lentivirus to astrocytes.

### *In-vivo* targeting of astrocytes from the CNS of mice

**Figure 2:**
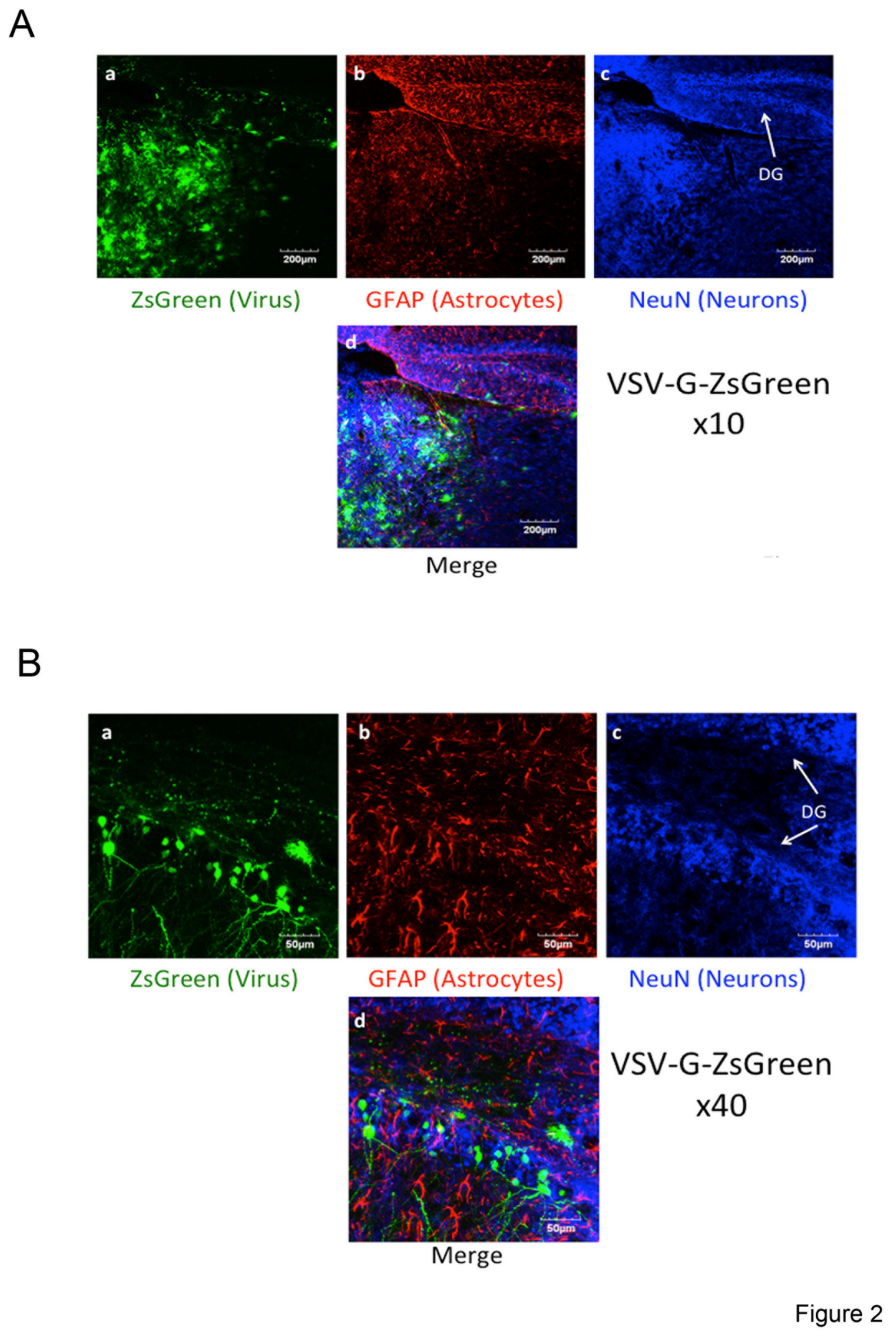
*In vivo* non-specific transduction of cells within the CNS by VSV-G pseudotyped lentivirus. Engineered lentiviruses were injected into the hippocampus or thalamus of 2-3 months old mice; 14 days post injection, brain slices were generated and subjected to immuno-fluorescent. Slices were stained with the indicated antibodies (GFAP for astrocytes and NeuN for neurons). Sections were then visualized by confocal microscopy to detect viral-mediated expression of ZsGreen, GFAP and NeuN staining. Images are the representative of n=3 mice. **A**. Efficient and non-specific broad spread of VSV-G pseudotyped lentiviruses in vivo. VSV-G-ZsGreen lentiviruses were injected into the brain of mice. 14 days post injection brain slices were generated and subjected to immuno-fluorescent staining. This analysis demonstrates the broad spread of engineered VSV-G pseudotyped lentiviruses and the non-specific pattern of their spread along the different areas and cell types in the brain. **panel a** - ZsGreen lentiviral expression; **panel b** - GFAP staining for astrocytes; **panel c** - NeuN staining for neurons; **panel d** - merged image for ZsGreen expression in neurons (NeuN) and astrocytes (GFAP). Indicated scale bar, 200µm (zoom x10). **B**. Higher magnification of brain sections that were transduced with VSV-G pseudotyped reporter lentiviruses (VSV-G-ZsGreen). Images show non-specific transduction of the different cell types in the hippocampus. **panel a** - ZsGreen lentiviral expression; **panel b** - GFAP staining for astrocytes; **panel c** - NeuN staining for neurons; **panel d** - merged image for ZsGreen expression in neurons (NeuN) and astrocytes (GFAP). Light blue staining indicates co-localization of NeuN and ZsGreen. Indicated scale bar, 50µm (zoom x40).

**Figure 3:**
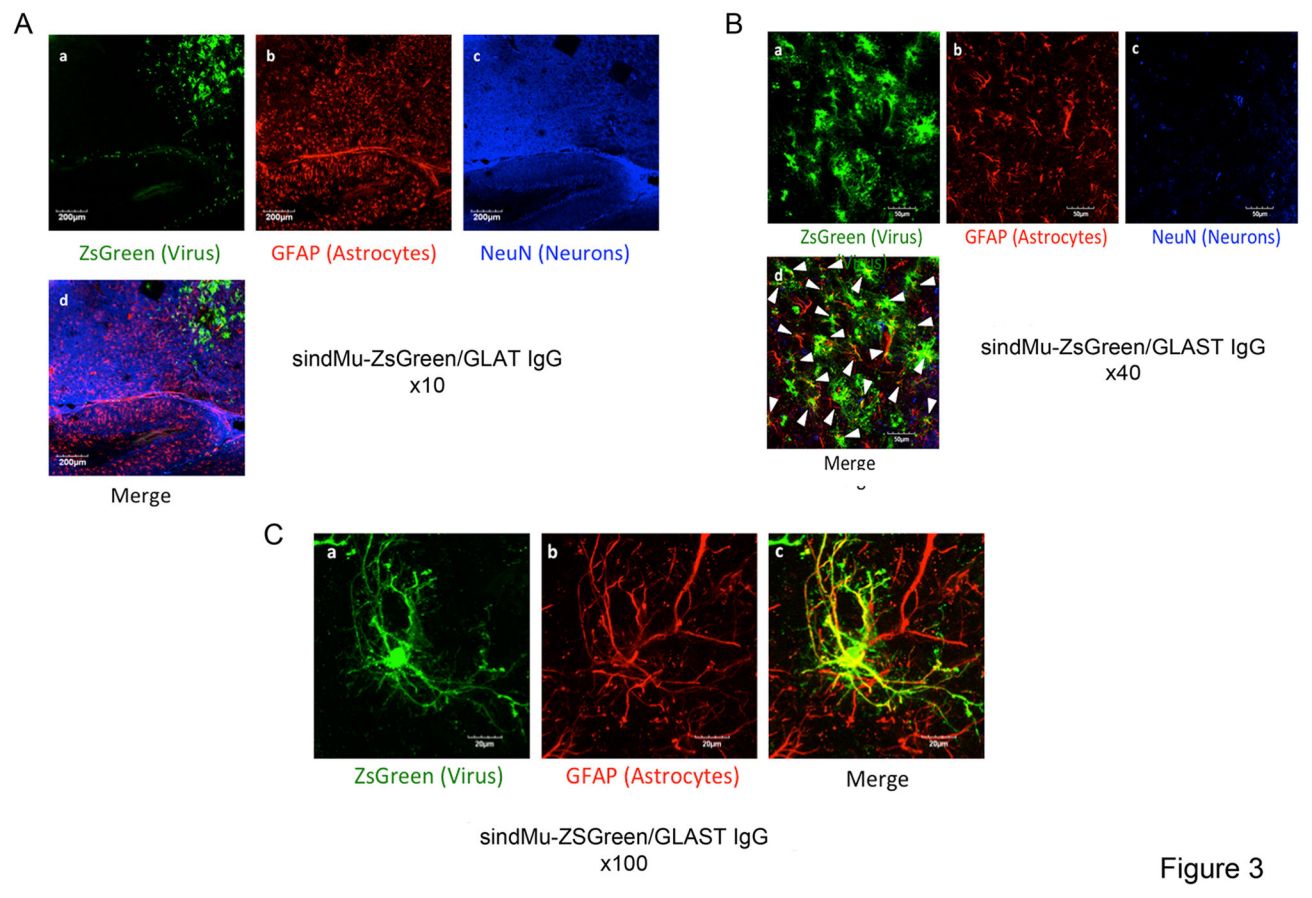
*In vivo* selective targeting of astrocytes by SindMu-ZsGreen/GLAST lentiviruses. **A**. Broad-field images of brain sections following transduction with sindMu-ZsGreen/GLAST IgG lentivirus. The figure shows defined areas in the brain and cell types that were transduced and expressed viral-mediated ZsGreen. **panel a** - ZsGreen lentiviral expression; **panel b** - GFAP staining for astrocytes; **panel c** - NeuN staining for neurons; **panel d** - merged image for ZsGreen expression in neurons (NeuN) and astrocytes (GFAP). Indicated scale bar, 200µm (zoom x10). **B**. Higher magnification of sindMu-ZsGreen/GLAST IgG transduction - engineered lentiviruses were injected into the hippocampus and thalamus of mice, and 14 days post injection brain slices were generated and stained with the appropriate antibodies. **panel a** - ZsGreen lentiviral expression; **panel b** - GFAP staining for astrocytes; **panel c** - NeuN staining for neurons; **panel d** – merged image for ZsGreen, GFAP and NeuN expression. Arrows present cells were GFAP and ZsGreen are co expressed. Indicated scale bar, 50µm (zoom x40). **C**. Single cell imaging of the above transductions with sindMu-ZsGreen/GLAST IgG lentiviruses. Analysis shows preferential targeting of astrocytes in the CNS of mice by SindMu-ZsGreen/GLAST-1 IgG. **panel a** - ZsGreen lentiviral expression; **panel b** - GFAP staining for astrocytes; **panel c** – merged image for ZsGreen and GFAP expression at the level on a single cell. Indicated scale bar - 20µm (zoom x100).

**Figure 4:**
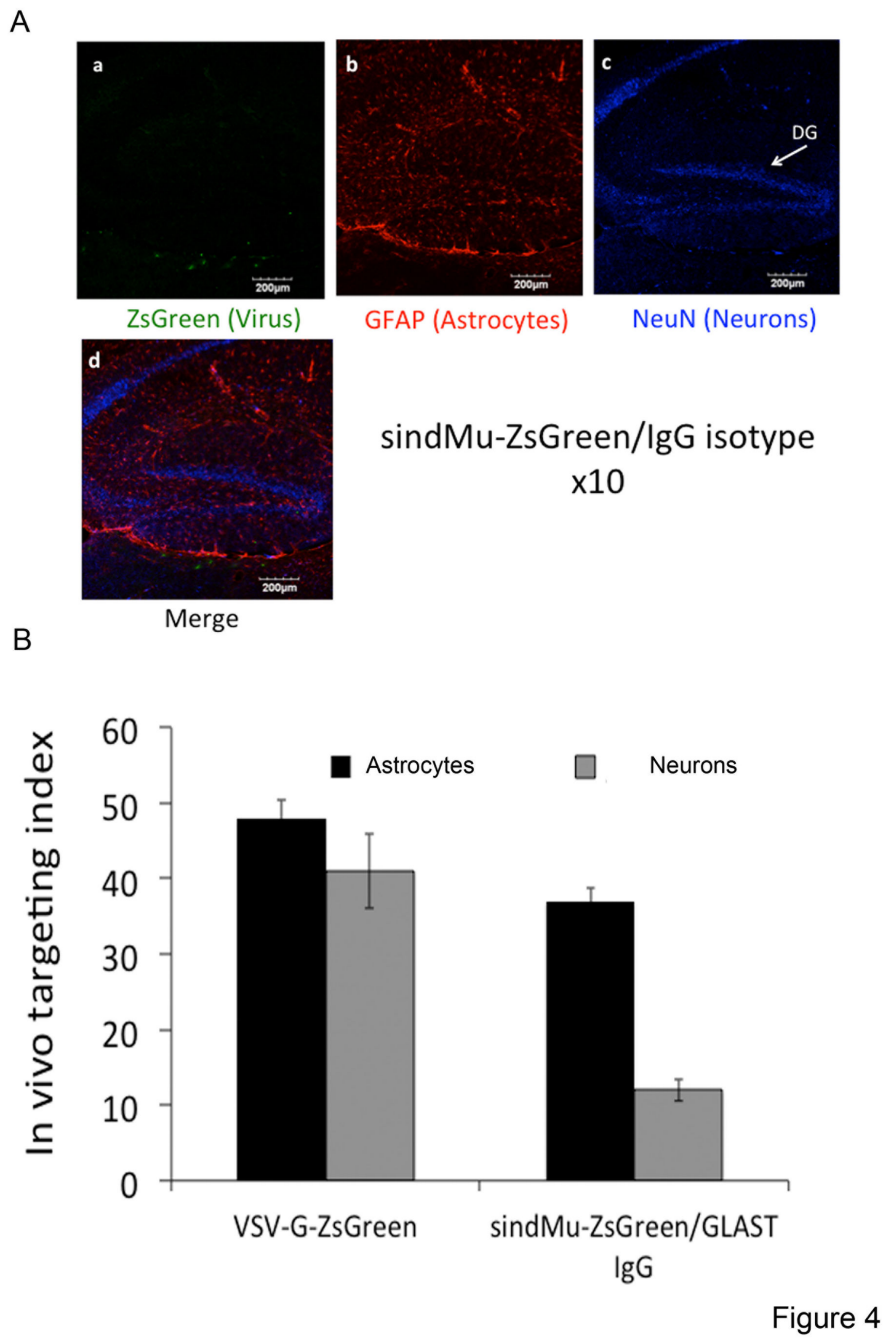
*In* *vivo* transduction with control sindMu-ZsGreen lentiviruses that display isotype IgG on their surface. To demonstrate specificity of the lentiviral targeting approach, *in* *vivo* brain sections were transduced with sindMu-ZsGreen lentiviruses that displayed isotype IgG on their surfaces (sindMu-ZsGreen/ IgG isotype). **image a** - ZsGreen lentiviral expression; **image b** - GFAP staining for astrocytes; **image c** – NeuN staining for neurons; **image d** – merged image for ZsGreen, GFAP and NeuN expression. Indicated scale bar - 50µm(zoom x40). **B**. Quantitation of in-vivo lentiviral targeting - Analysis of the relative percentage of ZsGreen-positive cells that also stained positively for NeuN or GFAP, following transduction with either VSV-G-ZsGreen or sindMu-ZsGreen/GLAST IgG recombinant lentiviruses. As shown, similar relative targeting index values for both astrocytes and neurons were observed when the VSV-G-ZsGreen was used. However, using sindMu-ZsGreen/GLAST IgG lentivirus, the NeuN-ZsGreen co-staining was significantly decreased, while the relative percentage of GFAP/ZsGreen-positive cells was significantly higher.

The ability of the lentiviral-targeting approach to serve as a tool to preferentially transduce astrocytes was further validated *in vivo*. Viral particles were stereotacticly injected into the dorsal hippocampus or the thalamus of 2-3-month-old mice. 14 days post injection, mice brain sections were generated and immuno-stained with monoclonal antibodies for an astrocyte marker, GFAP, or a neuronal, NeuN, marker. Brain sections were then analyzed by confocal microscopy for specific cell marker staining and for lentiviral-mediated ZsGreen expression (Figure 2). Initially, VSV-G pseudotyped lentiviruses that expressed ZsGreen (VSV-G-ZsGreen) were injected into the brains of mice in an attempt to demonstrate efficient, non-specific and broad *in vivo* spread of engineered sindMu pseudotyped viral particles (Figure 2A). As visualized, VSV-G-ZsGreen lentiviral spread was indeed efficient, and ZsGreen expression was detected in broad areas of the brain that stained positively for either GFAP or NeuN. At a higher resolution, VSV-G-ZsGreen exhibited a slight preference for neurons and was less prominent in areas of the brain that were enriched with astrocytes (Figure 2B; images d). To test the ability of the lentiviral targeting approach to preferentially target astrocytes *in-vivo*, sindMu-ZsGreen/GLAST IgG engineered lentiviruses (4 µl of 200ng p24/µl) were injected into the mouse brain at a rate of 0.5 µl/min (Figure 3A and 3B at higher magnification). As shown in *vivo*, sindMu-ZsGreen/GLAST IgG engineered lentiviruses demonstrated preferential transduction to areas of the brain that were enriched with astrocytes (Figure 3A; low magnification ×10). Furthermore, *in vivo*, sindMu-ZsGreen/GLAST IgG engineered lentivirus preferentially targeted astrocytes over neurons. At higher magnification of the confocal images, co-expression of GFAP and ZsGreen in the same cells was observed (Figure 3B image d at higher magnification and 3C image c at the level of a single cell). In contrast, lentiviral-mediated ZsGreen and NeuN co-expression was not detected (Figure 3B; image d; 3B image d). Importantly, cells that were also transduced with the control sindMu-ZsGreen lentiviruses that incorporated isotype IgG on their surfaces (sindMu-ZsGreen/ IgG isotype) did not show ZsGreen staining, confirming the inability of these control lentiviruses to mediate transduction without the IgG binding moiety (Figure 4A). Finally, *in vivo* lentiviral targeting of neurons (NeuN+) *versus* astrocytes (GFAP+) was quantified and compared for the sindMu-ZsGreen/GLAST IgG and VSV-G-ZsGreen lentiviruses. Image quantification analysis demonstrated that for the VSV-G-ZsGreen lentivirus, a similar targeting percentage index was observed for both neurons and astrocytes. In contrast, the relative percentage index of astrocytes was significantly higher when compared with the index value for neurons upon use of the sindMu-ZsGreen/GLAST IgG for transductions (Figure 4B). We conclude that the sindMu-ZsGreen/GLAST IgG engineered lentiviruses preferentially transduces astrocytes *in vivo*.

## Discussion

Selective gene transfer into cells of the CNS provides a powerful methodology for modulating gene function and understanding *in vivo* cell interactions in the brain under both normal and disease states. The main goal of this study was to validate recombinant lentiviruses that display GLAST IgG on their surface (sindMu-ZsGreen/GLAST IgG), as a tool for selective targeting of astrocytes. sindMu-ZsGreen/GLAST IgG lentiviruses expressed ZsGreen as a reporter transgene and were pseudotyped with a modified sindbis envelope (sindMu) that mediated only viral fusion. GLAST soluble IgG, incorporated on the surfaces of engineered sindMu lentiviruses, served as specific attachment moieties for astrocytes target cells. GLAST antigen is an ion transporter that is uniquely expressed on astrocytes. This lentiviral-mediated targeting approach is highly flexible, as different viral particles can incorporate different IgG on their surface, thus target different cells. Moreover, different cell types, can be marked simultaneously by a mixture of viral particles, each displaying a unique IgG targeting moiety. The current approach also overcomes the obstacle of low titers that is commonly observed in engineered lentiviruses. sindMu-ZsGreen/GLAST IgG exhibited high viral titers, that reached 5×10^8^ particles/ml, as measured on astrocyte target cells *in vitro*. These titers are comparable to those obtained with VSV-G pseudotyped lentiviruses. Importantly, resources that are used to generate these recombinant particles are low, and the only additional cost is the purchase of the targeting IgG.

Selective *in-vitro* transduction rates of astrocytes with the sindMu-ZsGreen/GLAST IgG lentiviruses were high and similar to those obtained with the VSV-G-ZsGreen lentiviruses (34% *versus* 40%, respectively; Figure 1). However, while the VSV-G-ZsGreen lentiviruses, were non-specific, as similar targeting rates were obtained for both astrocytes and microglia, the sindMu-ZsGreen/GLAST IgG exhibited relatively low tropism for microglia and higher tropism for astrocytes (Figure 1). Significantly, preferential tropism of the sindMu-ZsGreen/GLAST IgG lentiviruses for astrocytes was also demonstrated *in vivo*, following injection of recombinant lentivirus into the brains of mice. Indeed, selective transduction of sindMu-ZsGreen/GLAST IgG to GFAP positive astrocytes and preference over neurons that express NeuN was obtained (Figure 3). In contrast, VSV-G-ZsGreen lentiviruses efficiently transduced both cell types *in vivo*, with a slight lower preference for neurons (Figure 2A and 2B; Figure 4B). Viral engineered particles that displayed isotype IgG did not transduce any cells *in vivo* (Figure 4A). Despite the encouraging results that are presented in this work, current attempts in our lab are being performed in order to improve the targeting approach and obtain higher and cell-specific targeting rates, with lower non-specific cell transductions. First, the incorporation step of the specific targeting soluble IgG moieties on the virus surface is being optimized in order to increase the expression of IgG on the surface of the virus. Potential solutions will include expression of the IgG or scFvFc as part of the lentiviral transgene vector, thereby skipping the relatively inefficient incorporation step of soluble IgG on the surfaces of viral particles. Alternatively, incorporation of cell specific promoters, like the GFAP astrocyte specific promoter, into the lentiviral transgene vector should, also improves the selectivity of the targeting tool towards astrocytes. An additional caveat of the current approach is its dependence on the expression levels of the targeting antigen displayed on the surface of the infected cells. Although GLAST/EAAT1 expression is exclusively expressed on astrocytes, its expression levels can change and may even drop upon cell activation, or during neurodegenerative disorders. Some reports indicate that the expression of glutamate transporters in astrocytes and microglia are differentially regulated and expressed following nerve injury. Moreover, not only the expression but also the cellular localization of the glutamate transporters are changed under pathologic conditions [59,60]. As such, the use of GLAST or other astrocyte-specific transporters, like Glt1/EAAT2, may be limited. One way to overcome this limitation, mainly in animal models, is to inject the recombinant lentiviruses early in the disease development or prior to its induction. Significantly, unlike other viruses, the lentiviral approach enables long-term and stable gene expression, since the transgene is integrated into the host genome, and, then no longer affected by the levels of the surface-targeting antigen.

Recent attempts to exploit viral vectors as gene delivery vehicles into astrocytes have also included the use of AAV as a powerful tool for gene delivery specifically into astrocytes [61]. In that study, AAV vectors were used to target hippocampal astrocytes in APP/PS1 mice. In these vectors, the human GFAP specific promoter (Gfa2) drove the expression of VIVIT, a peptide that disrupts the inflammatory calcineurin/NFAT-signaling pathway, which plays a key role in astrocyte activation. A broad, highly specific and long-term transgene expression was found across the horizontal and longitudinal axes of the hippocampus, following a single intra-hippocampal injection. Almost no transgene expression was observed in neuronal cell bodies and microglia. Significantly, GFAP-VIVIT expression led to improved cognitive and synaptic functions, reduced glial activation and lower amyloid levels, thereby confirming the important role of astrocytes in Alzheimer’s disease [62].

Numerous studies have also stressed the promise of lentiviral-mediated targeting as an efficient tool for targeting cells of the CNS [63]. A recent study by Choi and colleagues demonstrated selective lentiviral transduction of a subset of inhibitory neurons in the cerebral cortex that express ErbB4, the neuregulin (NRG) receptor. In their study, both modified G-deleted rabies reporter virus and lentivirus were pseudotyped with the avian subgroup B envelope and incorporated a protein bridge composed of the avian viral receptor-TVB, fused to NRG. This approach can serve as a tool for further studies of NRG and ErbB receptors in brain circuits, as well as for further development of related bridge proteins to target gene expression to other specific cell types in complex tissues [64]. In yet another study, Cannon et al. [65] evaluated the *in-vivo* tropism of lentiviral particles for specific cells in the substantia nigra. Reporter lentiviral particles were pseudotyped with enveloped glycoproteins derived from VSV, MV, LCMV, or MuLV and injected into the substantia nigra of mice. This work demonstrated that viral delivery led to transgene expression in restricted and distinct cells. While VSV-G- and MV-pseudotyped lentiviruses targeted midbrain neurons, including a subset dopaminergic neurons in the substantia nigra, LCMV and MuLV exclusively transduced astrocytes. That study concluded that pseudotyped lentiviral vectors can be useful for experimental gene transfer to the specific area of the substantia nigra and for understanding the function of these cells in this specific area of the brain [65]. Importantly, Colin et al. used Mokola pseudotyped lentiviruses to demonstrate a shift of lentiviral transduction into astrocytes. However, they obtained rather low targeting efficiencies for astrocytes and high background transduction for neurons. They therefore had to combine the lentivirus targeting tool with the expression of miR124 to eliminate residual transduction of the lentivector into neurons. Nevertheless, background targeting of other cells, such as microglia, was not presented, emphasizing the advantage of this approach [66]. It will be interesting to combine our lentiviral-targeting tool with the expression of miR124 and further minimize non-specific transduction in microglia or neurons. Finally, a nanobody (Nb) display technology to target lentiviruses to dendritic cells and macrophages was recently applied by Goyvaerts et al. In addition to production of high titer lentiviruses, this approach demonstrated selective, nanobody-dependent transduction of mouse dendrites and macrophages both *in vitro* and *in vivo* [44]. We plan to apply this tool on cells of the CNS in combination with our system, generating viral particles that display target molecules in the form of nanobody.

Overall, the suggested lentiviral-mediated targeting approach joins the technologies described above and should be further tested for selective gene targeting of astrocytes and other cells in the CNS. It combines the advantages of lentiviral vectors with a high flexibility to preferentially target different cells types in the CNS. We anticipate that such a tool will be useful for simultaneous gene transfer and manipulation of genes expressed in different cells within the neurovascular unit. The developed methodology will also support tracing and imaging studies, during normal brain development, or following brain injury and in neurodegenerative diseases. As such, it could open a window for new approaches for research and treatments***.***
